# Leveraging Social Media to Increase Access to an Evidence-Based Diabetes Intervention Among Low-Income Chinese Immigrants: Protocol for a Pilot Randomized Controlled Trial

**DOI:** 10.2196/42554

**Published:** 2022-10-28

**Authors:** Lu Hu, Nadia Islam, Yiyang Zhang, Yun Shi, Huilin Li, Chan Wang, Mary Ann Sevick

**Affiliations:** 1 Center for Healthful Behavior Change Institute for Excellence in Health Equity NYU Langone Health New York, NY United States; 2 Department of Population Health NYU Grossman School of Medicine NYU Langone Health New York, NY United States; 3 Department of Medicine NYU Grossman School of Medicine NYU Langone Health New York, NY United States

**Keywords:** diabetes, health equity, immigrant health, mobile health, social media, messaging app, health education, education video, diabetes education, self-management

## Abstract

**Background:**

Type 2 diabetes (T2D) in Chinese Americans is a rising public health concern for the US health care system. The majority of Chinese Americans with T2D are foreign-born older immigrants and report limited English proficiency and health literacy. Multiple social determinants of health limit access to evidence-based diabetes interventions for underserved Chinese immigrants. A social media–based diabetes intervention may be feasible to reach this community.

**Objective:**

The purpose of the Chinese American Research and Education (CARE) study was to examine the potential efficacy of a social media–based intervention on glycemic control in Chinese Americans with T2D. Additionally, the study aimed to explore the potential effects of the intervention on psychosocial and behavioral factors involved in successful T2D management. In this report, we describe the design and protocol of the CARE trial.

**Methods:**

CARE was a pilot randomized controlled trial (RCT; n=60) of a 3-month intervention. Participants were randomized to one of two arms (n=30 each): wait-list control or CARE intervention. Each week, CARE intervention participants received two culturally and linguistically tailored diabetes self-management videos for a total of 12 weeks. Video links were delivered to participants via WeChat, a free and popular social media app among Chinese immigrants. In addition, CARE intervention participants received biweekly phone calls from the study’s community health workers to set goals related to T2D self-management and work on addressing goal-achievement barriers. Hemoglobin A_1c_ (HbA_1c_), self-efficacy, diabetes self-management behaviors, dietary intake, and physical activity were measured at baseline, 3 months, and 6 months. Piecewise linear mixed-effects modeling will be performed to examine intergroup differences in HbA_1c_ and psychosocial and behavioral outcomes.

**Results:**

This pilot RCT study was approved by the Institutional Review Board at NYU Grossman School of Medicine in March 2021. The first participant was enrolled in March 2021, and the recruitment goal (n=60) was met in March 2022. All data collection is expected to conclude by November 2022, with data analysis and study results ready for reporting by December 2023. Findings from this pilot RCT will further guide the team in planning a future large-scale study.

**Conclusions:**

This study will serve as an important first step in exploring scalable interventions to increase access to evidence-based diabetes interventions among underserved, low-income, immigrant populations. This has significant implications for chronic care in other high-risk immigrant groups, such as low-income Hispanic immigrants, who also bear a high T2D burden, face similar barriers to accessing diabetes programs, and report frequent social media use (eg, WhatsApp).

**Trial Registration:**

ClinicalTrials.gov NCT03557697; https://clinicaltrials.gov/ct2/show/NCT03557697

**International Registered Report Identifier (IRRID):**

DERR1-10.2196/42554

## Introduction

Type 2 diabetes (T2D), which accounts for 90% to 95% of diabetes cases in the United States [1], is a persistent public health issue and results in significant economic costs [2]. According to the latest statistics, diabetes affects 37.3 million Americans [3] and remains the seventh-leading cause of death in the nation [4]. T2D disproportionately affects racial and ethnic minorities, including Asian Americans [5,6]. Compared to non-Hispanic White people, Asian Americans bear a higher T2D burden [5,7]. Indeed, one report found that about one in two Chinese Americans in New York City (NYC) has diabetes or prediabetes [8]. Many of these individuals are low-income, foreign-born Chinese immigrants who speak little or no English [8].

Diabetes self-management education and support (DSMES) programs have been widely recognized as effective strategies to promote diabetes self-management and improve glycemic control [9,10]. Traditionally, DSMES involves multiple in-person office visits (eg, 6 to 12 1-hour-long group sessions) with a certified diabetes educator or health coach, who provides important education and counseling about diabetes self-management and lifestyle changes [9,10]. These programs are, however, difficult to access for Chinese immigrants with T2D because of the barriers related to social determinants of health. First, our prior studies showed that 80% of Chinese immigrants with T2D reported limited English proficiency [11,12], which has been shown to correlate with health care discrimination and miscommunication [13]. Previous studies also found that even with translation services, individuals who report Chinese to be their preferred language know less about diabetes and have higher hemoglobin A_1c_ (HbA_1c_) levels than those who prefer to speak English [14]. Second, the lack of cultural tailoring of existing evidence-based programs is another major barrier [15,16]. Most DSMES curricula are based on a western diet and culture, and dietary recommendations are often inconsistent with the dietary preferences of Chinese immigrants. Within such programs, Chinese immigrants find it difficult to initiate healthier dietary practices [15,17]. Third, these programs require traveling to a centralized location where counseling is delivered via multiple in-person sessions. Attending such programs can be challenging for low-income immigrants who face transportation barriers or have little-or-no sick leave [18]. Given the high T2D burden and the numerous barriers to accessing the DSMES programs, there is a pressing need to develop and test culturally tailored strategies to increase access to DSMES interventions for Chinese immigrants.

A social media–based strategy may be a viable solution to address the barriers mentioned above [19,20]. In earlier work, we found that in a sample of 91 older Chinese immigrants (mean age 70 years), most participants had smartphones and used a free social media app called WeChat to stay connected with family and friends in Mainland China or other parts of the United States [11]. The majority reported strong interest in receiving WeChat-based DSMES [11]. Building on these pilot data, we developed a 12-week culturally and linguistically tailored DSMES video program, consisting of two brief videos (5-10 minutes) per week sent to participants with T2D via WeChat, with biweekly phone calls to discuss the content and answer any questions they may have had [12]. Feasibility and acceptability of the intervention were assessed in a single-group pilot study (n=30), which demonstrated that over 90% of the videos were viewed, and 100% of participants were retained through the 12-week intervention period [12]. We also observed a reduction in HbA_1c_ and improvements in dietary behaviors and physical activity [12]. However, this was a single group pre- and posttest study design. To rigorously test this approach and control for potential confounders, we are conducting a pilot randomized controlled trial (RCT) to examine whether we can still observe the positive trends in improving health outcomes in Chinese immigrants with T2D.

The purpose of the Chinese American Research and Education (CARE) trial is to pilot-test the intervention in an RCT among Chinese Americans with T2D living in NYC. The main objective is to examine the potential efficacy of the CARE intervention for improving glycemic control. The secondary objective is to explore the potential effects of the CARE intervention on psychological and behavioral factors related to glycemic control. In this report, we describe the CARE trial study protocol.

## Methods

### Study Design

The CARE trial is a pilot RCT (n=60) with a 6-month duration. Participants were randomized to one of two arms (n=30 each): (1) wait-list control or (2) CARE intervention. HbA_1c_ levels, self-efficacy, diabetes self-management behaviors, dietary intake, and physical activity will be measured at baseline, 3 months, and 6 months.

### Ethics Approval

This study was approved by the NYU Grossman School of Medicine Institutional Review Board (protocol s18-00609) and was registered at ClinicalTrials.gov (NCT03557697).

### Eligibility Criteria

To be eligible for the study, participants must meet the following inclusion criteria:

1. Self-identify as a Chinese immigrant or Chinese American.
2. Be 18 to 70 years old.
3. Be able to speak and understand Mandarin.
4. Self-report a diagnosis of T2D.
5. Have a baseline HbA_1c_ of 7% or greater.
6. Have experience using WeChat or text messages.
7. Be willing to receive WeChat or text messages regarding T2D management.
8. Express willingness and confidence in their ability to watch two diabetes videos each week for a total of 12 weeks.
9. Express motivation to make lifestyle changes to control their diabetes.
10. Be willing to wear an ActiGraph accelerometer for 8 days.

Individuals are excluded from participation if they do any of the following:

1. Are unable or unwilling to provide verbal consent.
2. Are unable to participate meaningfully in the intervention (eg, uncorrected sight and hearing impairment).
3. Are unwilling to accept their randomization assignment.
4. Are pregnant or plan to become pregnant in the next 6 months, or become pregnant during the study.
5. Are breastfeeding.
6. Live in a facility or other health care setting where they have no control over diabetes self-management.

### Recruitment

#### Overview

The study employed multipronged recruitment strategies within several NYC health care facilities, including the Charles B. Wang Community Health Center (CBWCHC), private primary care providers (PCPs), and NYU Langone Health (NYULH) and affiliated practices.

CBWCHC is a federally qualified health center with locations in both Manhattan’s Chinatown and Queens Flushing’s Chinatown. CBWCHC is one of the largest and leading community centers in NYC, having established a trusting relationship with the Chinese American community. Given that many Chinese immigrants seek diabetes care from their PCPs, we also worked with several PCP offices to recruit. In addition, NYU Langone Brooklyn Family Health Center is also a federally qualified health center and serves a large number of low-income Chinese and Hispanic immigrants in the Brooklyn Sunset Park area. We recruited participants from these clinic partners using three methods: posters, direct referral by providers, and electronic medical record Epic search and DataCore.

#### Posters

Posters were placed in the mentioned health care facilities’ waiting and examination rooms. Posters listed a contact telephone number that patients can call if interested in enrolling. Patients who self-referred were screened for eligibility.

#### Direct Referral by Providers

CBWCHC, NYU Langone Brooklyn site providers, and private practice PCPs approached patients who were potentially eligible for the study and solicited their interest in the study. When patients expressed verbal interest in study participation, health care providers shared the patient’s contact information with the study staff, or patients were advised to self-refer using the information provided on the study poster. A study staff member called the patient and, with the patient’s verbal consent, the staff member conducted a telephone screening to confirm eligibility prior to study enrollment.

#### Electronic Medical Record Epic Search and DataCore

We worked through the NYULH DataCore—a resource that provides the NYULH research community with clinical data from the NYULH electronic medical record system and ancillary clinical systems—to recruit participants from NYU Langone Manhattan, Brooklyn, and Long Island campuses. The Epic electronic medical record was queried to identify and generate a report of potentially eligible patients, including their name, sex, date of birth, address, phone number, weight, height, BMI, name of PCP, and the date and result of their most recent HbA_1c_ test. Potentially eligible individuals were sent a letter via US postal mail describing the study and were provided with directions for opting out of future recruitment calls. Study staff contacted those not opting out to describe the study and, if interested, screened them for eligibility.

### Overview of the Intervention

We are leveraging the DSMES intervention used in the Enhancing Adherence in Type 2 Diabetes (ENHANCE) trial, which has been shown to be effective for decreasing HbA_1c_ levels in a highly educated, predominately non-Hispanic White population [21]. Similar to the ENHANCE intervention, the CARE intervention is based on the widely used Social Cognitive Theory (SCT), which posits that self-efficacy is an important determinant of the performance of behavior and is affected by four major sources of information: mastery experiences, social modeling, verbal persuasion, and physiological states [22,23]. To promote mastery experience, intervention videos focused on encouraging participants to set incremental, easily achievable goals, and self-evaluate progress toward goals. Participants were counseled about the use of self-reward for goal achievement. Videos also involved training in problem-solving around common barriers to self-management. We operationalized social modeling with videos showing a Chinese patient sharing a similar immigration background who has been successful in managing T2D. We used verbal persuasion to support behavior change. We guided participants to recognize the physiologic benefits they would experience as a result of dietary changes and increased physical activity (eg, better sleep and better glucose control). In addition to the SCT-based content, we delivered educational videos about medication adherence, glucose monitoring, stress management, healthy eating and cooking, and tips to engage in physical activity.

The intervention adaptation was guided by the Cultural Adaptation Model [24] and the Ecological Validity Model (EVM) [25]. According to these models, the target community should be actively engaged throughout the adaptation process. During the first 2 years, we conducted extensive formative work to culturally adapt the ENHANCE intervention for Chinese immigrants. For example, we convened a community advisory board, which consisted of a primary care doctor, a diabetes educator, a nurse, and a patient with T2D, all of whom were Chinese immigrants or Chinese Americans and understood Chinese culture. We shared the outline of the intervention content and specific aspects of cultural adaptation (Table 1). Video prototypes were reviewed by members of the community advisory board for necessary modifications prior to their implementation.

The EVM adaptation model suggests considering the following eight domains for adaptation: language, person, metaphors, content, concepts, goal, method, and context [25]. For *language*, we translated the intervention materials into Mandarin Chinese. Whereas there are other dialects spoken by Chinese individuals, Mandarin is the official language of China and is understood by most people [26]. With regard to the *person* domain, all CARE team members are bilingual and bicultural members of the Chinese community. For the *metaphors* domain, we incorporated culturally relevant metaphors into the intervention content as appropriate. For the *content* and *concepts* domains, we tailored the T2D-related content and concepts based on Chinese culture and norms. For instance, we provided recommendations for healthy eating during traditional Chinese festivals. We also created a video demonstration of healthy grocery shopping at Chinese supermarkets. In the *goal* and *method* domains, the EVM advises delivering interventions using methods that are mutually agreeable and culturally acceptable. For example, rather than dictating study goals, patients were encouraged to set their own goals for participation. In addition, we used WeChat to deliver our intervention, a platform with which many Chinese immigrants are familiar. Lastly, with regard to the *context*, we provided information on local health care resources and Chinese grocery stores.

**Table 1:** Intervention content and cultural adaptation.

Week	Intervention component (educational + behavioral content)	Cultural adaptation
1	Overview of diabetes, self-management, and goals for life	T2D^a^ risks in the context of Chinese culture and norms
2	T2D diet, healthy eating, and portion control (part 1); setting goals	T2D diet in the context of Chinese culture and norms
3	T2D diet, healthy eating, and portion control (part 2); self-reward	T2D diet in the context of Chinese culture and norms
4	Medication management; social support	Common barriers to taking medication among Chinese patients with T2D and strategies
5	Glucose self-monitoring; problem-solving model; problem-solving: barriers and setbacks	Local resources for obtaining free testing supplies
6	Exercise and diabetes; problem-solving: behavioral triggers	Misconceptions about exercise in Chinese culture and exercise examples popular in Chinese culture (eg, Tai Chi)
7	Building muscles with strength training; problem-solving: emotional eating	Misconceptions about strength training in Chinese culture and tips for starting strength training
8	Grocery shopping at a Chinese supermarket; problem-solving: cravings for white rice	A grocery shopping video at a Chinese supermarket in Chinatown
9	Stress and T2D; problem-solving: eliminating negative self-talk	Common causes of stress among Chinese patients with T2D and ways to cope
10	Chinese holidays and dining out; problem-solving: anticipating high-risk situations	Tips for healthy eating during Chinese festivals
11	Attending doctor appointments; problem-solving: lapses and relapse	Common barriers to seeking care among Chinese patients with T2D; local resources for low-income Chinese patients with T2D
12	Navigating the US health care system; problem-solving: coping with lapses and setting new goals	Local resources for low-income Chinese patients with T2D

^a^T2D: type 2 diabetes.

### Randomization and Intervention Groups

### Overview

The study’s bilingual community health workers (CHWs) called patients and provided more details about the study. If the patient expressed interest in participation, the study CHW administered several screening questions. After eligibility was determined and verbal consent was obtained, a baseline survey was administrated over the phone, after which participants were randomized via a computer-generated randomization scheme with equal allocation to one of the two groups: wait-list control or CARE intervention.

#### Wait-list Control Group

Control group participants continued to receive the standard of care for T2D from their providers during our study. At the end of the study, all study videos will be offered to participants in the control group via WeChat links.

#### CARE Intervention

Participants in the intervention group received the standard of care, plus brief prerecorded videos, which included both educational and SCT-based behavioral content. Two culturally and linguistically tailored video links were sent each week via WeChat or regular text messages for a total of 12 weeks. Videos were 5 to 10 minutes in duration. WeChat and text messages were used only for video delivery. All other communication occurred via regular phone calls made from an NYULH-provided study phone. In particular, video delivery was supplemented by biweekly phone calls from the study CHW to review the video content, clarify questions, guide participants in setting goals, review progress toward goal achievement, and problem-solve any barriers or problems they encountered.

### Procedures

The study involves measurements at baseline, 3 months, and 6 months. Participants will be paid US $30 for each completed measurement. Due to COVID-19, all data will be collected via telephone. To offset data plan expenditures incurred from watching intervention videos using their mobile phones, participants from both groups will be paid US $5 per week for a maximum total payment of US $60.

### Measures

#### Primary Outcome

HbA_1c_ level is the primary outcome. As part of usual care, patients with T2D receive an HbA_1c_ blood test at their doctors’ offices every 3 to 6 months. To minimize participant burden and possible COVID-19 exposure, no additional laboratory tests are required. Rather, HbA_1c_ results will be extracted from participants’ electronic medical records.

#### Secondary Outcomes

**Figure 1:**
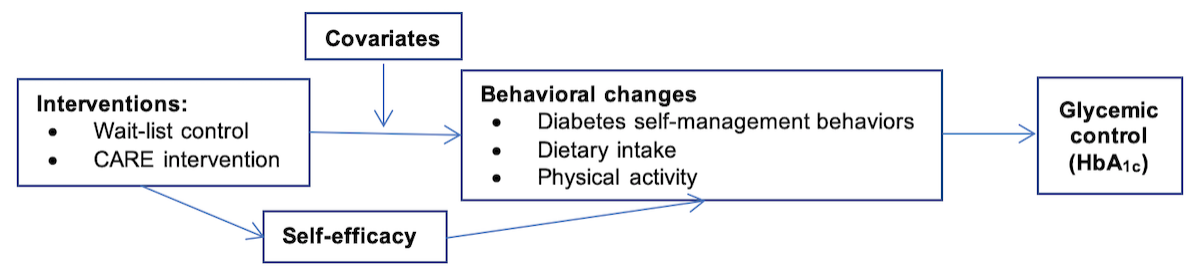
Conceptual model. CARE: Chinese American Research and Education; HbA_1c_: hemoglobin A_1c_.

Consistent with our conceptual model (Figure 1), we will measure self-efficacy, diabetes self-management behaviors, dietary intake, and physical activity at each time point (ie, baseline, 3 months, and 6 months).

##### Self-efficacy

We used the Stanford Diabetes Self-Efficacy Scale [27] to measure participants’ confidence in managing T2D. This instrument contains eight items and asks participants to rate their confidence level in performing specific self-management behaviors, using a 10-point Likert scale ranging from 1 (not at all confident) to 10 (totally confident).

##### Diabetes Self-Management Behaviors

We administered the Summary of Diabetes Self-Care Activities measure [28] to assess participants’ adherence to diabetes self-management behaviors. This scale consists of 13 items and asks participants to describe their diabetes self-care activities over the past 7 days. It assesses participants’ adherence to several key diabetes self-management behaviors, including diet, exercise, self-monitoring of blood glucose, foot care, and medication adherence.

##### Dietary Intake

Informed by a previous study among Chinese Americans [29], we used the adapted Mediterranean Dietary Screener [30] to estimate participants’ dietary intake behaviors at baseline, 3 months, and 6 months. This questionnaire asks for participants’ daily average intake of fruit, vegetables, refined grains, whole grains, sugary drinks, and potatoes over the past 30 days.

##### Physical Activity

We used the International Physical Activity Questionnaire [31] short version to assess the frequency and duration of various physical activities undertaken by adults over the past 7 days. This questionnaire will provide an estimate of the number of minutes per week participants engage in each category of physical activity (eg, vigorous, moderate, and mild intensity, as well as sedentary activity). We also used ActiGraph activity monitors to objectively measure physical activity and sleep. We mailed the device to participants once they were eligible for the study. Participants wore the ActiGraph for 8 days and returned it to the investigators using a prepaid envelope.

### Covariates: Sociodemographic and Health Characteristics

A sociodemographic questionnaire was used to collect basic information about the participant, such as age, gender, education, income, duration of residence in the United States, health insurance, duration of T2D, and medical history.

### Data Analysis

An intention-to-treat approach will be employed. We will also conduct a detailed descriptive analysis of all the data collected in the study. To establish the proof of concept regarding the efficacy of the CARE intervention for glycemic control among Chinese immigrants with T2D, we will test whether the two groups are comparable on baseline sociodemographic and health characteristics. If significant differences are found, we will include the variables as covariates in the models. We will fit the longitudinally collected HbA_1c_ measurement using piecewise linear mixed-effects models to compare the group difference in the HbA_1c_ level’s changing trend over different periods, adjusting for the covariates as needed.

To explore the potential effects of the CARE intervention on psychosocial and behavioral factors in glycemic control, we will analyze the collected secondary outcomes. For continuous secondary outcome variables (ie, self-efficacy, adherence to diabetes self-management behaviors, physical activity, and dietary intake), piecewise linear mixed-effects modeling will be used to examine the group difference in each outcome’s changing trend from baseline to 3 months and from 3 to 6 months.

### Sample Size Justification

According to Whitehead et al [32], to detect a small, standardized effect size of 0.2 in the primary outcome of HbA_1c_ with 80% power at a 5% significance level, a sample size of 50 is needed for a pilot RCT. Given the data from prior studies among Chinese Americans [33-35], we estimated an attrition rate of 15%. Thus, we planned to recruit a total of 60 participants, with 30 in each group, for this pilot RCT. With regard to secondary outcomes, given the pilot nature of this trial, we are not adequately powered to detect significant differences in self-efficacy, adherence to diabetes management behaviors, dietary intake, or physical activity.

## Results

This pilot RCT is part of a 5-year career development K99/R00 award funded by the National Institutes of Health (NIH) from 2018 to 2023. The first 2 years of this career award (K99 phase) were devoted to formative research, including a qualitative study to understand barriers faced by Chinese immigrants with T2D and a single-group study to develop and test the feasibility of the social media–based CARE intervention [12]. The R00 phase started in June 2020, and the goal is to conduct a pilot RCT to examine the potential efficacy of the CARE intervention. This pilot RCT was launched in March 2021, with the first participant enrollment in March 2021. A total of 60 participants have been enrolled and were computer-randomized into two groups in March 2022. The 3-month assessment for all participants has been completed. Data collection for the 6-month assessment is expected to conclude in November 2022. Data analysis and final study results are expected to be reported by December 2023.

## Discussion

### Overview

This report describes the protocol and design of a pilot RCT of a social media–based diabetes intervention among Chinese immigrants with T2D. We hypothesize that this social media–based strategy is a promising medium to increase Chinese immigrants’ self-efficacy and knowledge about their T2D, improve their dietary and physical activity behaviors, and ultimately improve their glycemic control. Participants in the intervention group are anticipated to have a greater reduction in HbA_1c_, to be more confident in managing their diabetes, to consume healthier diets, and to be more active in physical activities than those in the control group.

It is well documented that T2D disproportionately affects racial and ethnic minorities [36], who face many barriers to accessing evidence-based diabetes interventions, barriers that are related to social determinants of health; these interventions are usually delivered with multiple in-person sessions [37,38]. Leveraging a social media platform that the underserved communities are familiar with is a convenient and scalable approach to enhancing their access to DSMES [11,12]. If ultimately found to be effective, CARE could easily be delivered to patients with T2D residing in other US locations that have large Chinese populations. CARE may also serve as a program model for reducing disparities in other high-risk populations that also bear a high T2D burden and face many social and health-related barriers to care, including African, Native, and Hispanic Americans and adults living in rural areas [37,39].

### Strengths, Limitations, and Future Directions

CARE is a proof-of-concept randomized trial, the sample size is relatively small, and we are not adequately powered to detect statistical significances between groups for the secondary outcomes. However, informed by the NIH Stage Model for Behavioral Intervention Development [40], large investments in full-scale RCTs should be informed by pilot studies. Lessons learned in this pilot trial will also help us to plan the larger trial with regard to recruitment strategies, intervention videos, and survey questions. In addition, given the limited resources, we were only able to develop 24 videos in Mandarin Chinese. Therefore, for those who can only understand Cantonese or other dialects, they are not eligible to join the program. Yet, based on the most recent data, many Chinese immigrants who speak a different dialect are still able to understand Mandarin Chinese [26]. In addition, the study participants were only recruited from the NYC metropolitan area, and results may not be generalizable to the Chinese populations living in the Midwest where access to culturally tailored care may be even more limited.

Nonetheless, this study represents an initial step to exploring potential scalable strategies to increase access to evidence-based interventions among underserved, low-income, minority populations. If the results are promising, this may serve as an important milestone in considering how to deliver culturally and linguistically tailored interventions in underserved immigrant populations. Findings from this study will also serve as the foundation for a large full-scale RCT to test the program in a broad population.

### Dissemination Plan

We will disseminate research findings through presentations at regional (eg, the Annual Health Disparity Symposium at NYU and the NYU Asian American Pacific Islander Health conference), national (eg, the American Diabetes Association Annual Symposium), and international meetings and conferences (eg, the International Conference on Behavioral Medicine). We will also publish findings in peer-reviewed journals. In addition, we will work closely with our partners to identify strategies to disseminate study findings to the Chinese American community. This can include patient forums held at community locations (eg, parks and community centers), newsletters mailed to study participants, or a one-page brief summary of research findings. We will also post study findings via social media platforms (eg, Twitter and WeChat).

## Appendix

Multimedia Appendix 1 Peer-review report reviewed by: National Institute on Minority Health and Health Disparities Special Emphasis Panel - NIH Pathway to Independence Award (National Institutes of Health, USA).

## Abbreviations

CARE: Chinese American Research and Education
CBWCHC: Charles B. Wang Community Health Center
CHW: community health worker
DSMES: diabetes self-management education and support
ENHANCE: Enhancing Adherence in Type 2 Diabetes
EVM: Ecological Validity Model
HbA_1c_: hemoglobin A_1c_
NIH: National Institutes of Health
NYC: New York City
NYULH: NYU Langone Health
PCP: primary care provider
RCT: randomized controlled trial
SCT: Social Cognitive Theory
T2D: type 2 diabetes

## Data Availability

The data sets generated or analyzed during this study are available from the corresponding author upon reasonable request.
