# Supplementary material for: Leveraging Social Media to Increase Access to an Evidence-Based Diabetes Intervention Among Low-Income Chinese Immigrants: Protocol for a Pilot Randomized Controlled Trial
Source: JMIR Res Protoc. 2022 Oct 28;11(10):e42554. doi: 10.2196/42554 (PMC9652737; doi:10.2196/42554)
Supplement: Multimedia Appendix 1 [file resprot_v11i10e42554_app1.pdf]

SUMMARY STATEMENT
(Privileged Communication)

PROGRAM CONTACT:
Jennifer Alvidrez
301-594-9567
alvidrezjl@mail.nih.gov

Release Date: 03/21/2018
Revised Date:

Application Number: 1 K99 MD012811-01

Principal Investigator

HU, LU

Applicant Organization: NEW YORK UNIVERSITY SCHOOL OF MEDICINE

Review Group: ZMD1 XLN (M1)
National Institute on Minority Health and Health Disparities Special Emphasis Panel
NIH Pathway to Independence Award (Parent K99/R00)

Meeting Date: 03/01/2018
Council: MAY 2018
Requested Start: 07/01/2018

RFA/PA: PA16-193
PCC: CHS02AL
Dual PCC: RAJ DUAL
Dual IC(s): DK

Project Title: A Mobile Health Intervention to Reduce Diabetes Disparities in Chinese Americans

SRG Action: Impact Score:24
Next Steps: Visit https://grants.nih.gov/grants/next_steps.htm
Human Subjects: 30-Human subjects involved - Certified, no SRG concerns
Animal Subjects: 10-No live vertebrate animals involved for competing appl.
Gender: 1A-Both genders, scientifically acceptable
Minority: 2A-Only minorities, scientifically acceptable
Children: 3A-No children included, scientifically acceptable

Project Year	Direct Costs Requested	Estimated Total Cost
1	96,481	97,950
2	98,667	100,169
3	249,000	252,791
4	249,000	252,791
5	249,000	252,791
TOTAL	942,148	956,493

ADMINISTRATIVE BUDGET NOTE: The budget shown is the requested budget and has not been adjusted to reflect any recommendations made by reviewers. If an award is planned, the costs will be calculated by Institute grants management staff based on the recommendations outlined below in the COMMITTEE BUDGET RECOMMENDATIONS section.

1K99MD012811-01 Hu, Lu

RESUME AND SUMMARY OF DISCUSSION: This application is submitted in response to PA16-193, NIH Pathway to Independence Award (Parent K99/R00). The purpose of this project is to develop a linguistically and culturally tailored mobile Health (mHealth) intervention to reduce health disparities in underserved Chinese Americans with type 2 diabetes (T2D). The panel members are all quite enthusiastic about the critical area of health disparities research among Chinese Americans and the focus to develop an advanced mHealth tool. Specific strengths of this application included the strong research record and stellar mentoring team as well as the significant resources and support. The candidate is also perceived to be strong. Research plan is ambitious, but achievable as the support of mentoring team is substantial and candidate has preliminary studies and strong collaborations to execute work in the timeline. However, some concerns are raised regarding the community-based participatory research (CBPR) plan for the lack of details on how CBPR and the participatory aspects will inform each step of the research and how CBPR-related work will be translated into the modifications to the short message service (SMS) intervention. The CBPR approach, which is primarily for recruitment, is an inadequate use of CBPR principles in the field, although mentorship with Drs. Trinh-Shervin and Ogedegbe will help. The training plan for CBPR seems solid and will improve CBPR research plan. Overall, the application is rated in the excellent to outstanding range and could have high impact for the affected population.

DESCRIPTION (provided by applicant): Chinese Americans are one of the fastest growing immigrant groups in the US, who suffer disproportionately high type 2 diabetes (T2D) burden and have poorly controlled T2D. Despite the well-documented T2D disparities in this minority group, limited work has been conducted to improve health outcomes in Chinese Americans. The goal of this Pathway to Independence Award (K99/R00) is to expedite the candidate's transition to an independent investigator who possesses focused expertise in development and evaluation of culturally and linguistically tailored and sustainable interventions to reduce T2D disparities in Chinese Americans. In the K99 phase of this award, the candidate will obtain critical training needed to accomplish this goal and will develop a short message service (SMS) intervention to improve T2D management in Chinese Americans. In the R00 phase, the candidate will utilize acquired skills to conduct a pilot randomized controlled trial to examine the potential efficacy of the SMS intervention. In the K99 phase, the candidate will also conduct pilot work to develop linguistically and culturally tailored SMS intervention content and to refine the intervention to be tested in the R00 phase. More specifically, the aims are to 1) characterize barriers and facilitators of glycemic control in Chinese Americans with T2D (Aim 1a); 2) develop culturally and linguistically tailored SMS intervention content (Aim 1b); and 3) assess the feasibility and acceptability of the SMS intervention in a pre-, post-test study (Aim 1c). In the R00 phase, the candidate will refine the SMS intervention based on the K99 pilot data and evaluate the proof-of-concept regarding its efficacy in a pilot randomized controlled trial among 66 Chinese Americans with T2D (Aim 2). Participants will be randomized to one of 2 arms (n=33 each): 1) wait-list control and 2) SMS intervention. Both groups will continue to receive standard of care treatment for their T2D. The SMS group will receive brief lifestyle counseling videos via SMS links. At the end of the study, the wait-list control group will be provided the opportunity to receive the SMS-based counseling videos. Measurements will be obtained at baseline, 3, and 6 months. The primary outcome is HbA1c and secondary outcomes include self-efficacy, diabetes self-management behaviors, dietary intake and physical activity behaviors. Linear mixed modeling will be used to examine the group and group by time interaction effects between the SMS intervention and wait-list control group. Findings from this R00 study will inform a larger full-scale R01 efficacy trial of the SMS intervention, and ultimately, establish the candidate's program of research focused on developing and testing sustainable interventions to reduce disparities in chronic disease outcomes in Chinese Americans. This project can serve as a program model for other chronic disease interventions in Chinese Americans that require lifestyle modification (e.g., prediabetes, hypertension), or for disparities research in other high-risk immigrant populations (e.g., South Asians, Hispanic Americans).

PUBLIC HEALTH RELEVANCE
Type 2 diabetes (T2D) in Chinese Americans is a rising public health concern for the US health care system as this ethnic minority group has a high prevalence of T2D, continues to rapidly grow in the US, demonstrates suboptimal self-management behaviors, and has poorly controlled T2D. Given the high economic and societal burden of T2D and rapid population growth in Chinese Americans, there is a pressing need for research to develop effective and sustainable interventions to address T2D burden in this population. This proposed study seeks to reduce disparities and improve health outcomes in underserved Chinese Americans with T2D through a linguistically and culturally tailored mobile health intervention.

CRITIQUES: The critiques below were prepared by the reviewers assigned to this application. These commentaries do not necessarily reflect the position of the authors at the close of the group discussion, nor the final majority opinion of the group, although reviewers are asked to amend their critiques if their position changed during the discussion. The resume and summary of discussion, together with the sections at the end of the summary statement, which summarize the committee's final opinion on the use of human subjects, the inclusion of women, minorities, and children, the use of vertebrate animals, and budget are the authoritative representation of the final outcome of group discussion. If there is any discrepancy between the peer reviewers' commentaries and the numerical score on the face page of this summary statement, the numerical score should be considered the most accurate representation of the final outcome of the group discussion.

CRITIQUE 1

Candidate: 1
Career Development Plan/Career Goals /Plan to Provide Mentoring: 2
Research Plan: 4
Mentor(s), Co-Mentor(s), Consultant(s), Collaborator(s): 1
Environment Commitment to the Candidate: 1

Overall Impact:

The application, submitted by a productive and accomplished postdoctoral researcher with a well-balanced and accomplished mentorship team, responds to PA-16-193. The application proposes to reduce disparities and improve health outcomes in underserved Chinese Americans with T2D through linguistically and culturally tailored mobile health intervention. This is highly significant research questions that will inform population health interventions. The candidate has a relevant prior training and demonstrated academic productivity and has a comprehensive and strong mentorship team. The scientific premise of the research is strong. The research builds upon the candidate's doctoral thesis and post-doctoral research in diabetes disparities. The proposed K99 phase will provide training needed to accomplish this goal and will develop a short message service (SMS) intervention to improve T2D management in Chinese Americans, and the R00 phase will utilize acquired skills to conduct a pilot randomized controlled trial to examine the potential efficacy of the SMS intervention. This is a complex and ambitious intervention study that will ultimately inform and efficacy trial. A unique aspect of the training plain includes CBPR methodology and expertise working with Limited English Proficient Population and developing and designing tailored behavioral intervention trials. Challenges include the exploration of both SMS and video based counseling, and multiple intervention elements that could impact the feasibility and acceptability and ultimately influence the ability to carry out the second aim of the study. The mentorship team has high level expertise in these areas.

1. Candidate:

Strengths

- Major: Strong track record of academic productivity and scholarship with over 17 publications.
- Major: Relevant background and research training and an outstanding research and scholarship trajectory to date.
- Minor: Senior fellow since 2016 (completion of PhD).
- Minor: Outstanding letters of recommendation.
- Moderate: past funding for preliminary study from the Aetna Foundation Grant on Mobile Insulin Titration Intervention.

Weaknesses

- None identified.

2. Career Development Plan/Career Goals & Objectives/Plan to Provide Mentoring:

Strengths

- Major: Focus on three relevant areas of career development: causal inference methodologies, study of stress and CVD and racial/ethnic health disparities, with significant opportunities for extending methodological training and understanding of theoretical frameworks and analytic approaches in each area.
- Major: Clear communication and meeting plan for each mentor.
- Moderate: Achievable course of study given the timeline. Clear outline of coursework and research timelines.

Weaknesses

- Moderate: There is a single course on biological measurements in epidemiologic research and it would improve the career development plan to outline several specific goals of her training in the strengths and limitations of biomarker use in research (for example, collection, storage, analysis, error, etc.).
- Moderate: The supervision plan lacks adequate specificity regarding responsibilities of each mentor regarding support for the specific dimensions of the research plan.
- Minor: Guidance on analyzing previously collected biomarker data is included as consultation with the CSDE Biodemography Lab, this should perhaps be an expanded area of the career development plan due to the relevance of understanding data collection methods and storage to the analysis and interpretation of findings.

3. Research Plan:

Strengths

- Major: Research focuses on a significant and under investigated area of work: the association between precarious employment and CVD and obesity and the potential contribution to racial/ethnic disparities in health.
- Major: Strong letter of commitment from Community partners and budgeted support for advisory committee.
- Moderate: Use of the SCT theoretical framework and based on the success of the ENHANCE trail.

- Moderate: plan for regular Community Advisory Board involvement, demonstrated community engagement, limited participatory demonstration however.

Weaknesses

- Major: The R00 phase primary aims 2a and 2b require success, high feasibility and acceptability of the SMS intervention in order to proceed.
- Moderate: Text messaging interventions focused on health promotion around diabetes have been understudied and there is limited evidence demonstrating effectiveness. Given multiple variables: population characteristics, health literacy, language, affordability, it is a higher risk study.
- Moderate: The video-counseling dimension of the intervention adds an additional dimension to the SMS and is not well discussed in the analysis plan or aims.
- Sex is inadequately discussed in the analysis plan.

4. Mentor(s), Co-Mentor(s), Consultant(s), Collaborator(s):

Strengths

- Major: Primary mentor and co-mentors have an excellent track record of junior investigator training and support.
- Major: Outstanding mentorship team that has demonstrated expertise for the outlined course of study: Dr. Sevick the primary mentor has expertise in theory-based video counseling for T2D management and mHealth interventions and Dr. Trinh-Shevrin has extensive CBPR expertise and engagement with Asian American Health in the population area of focus, and Dr. Ogedegbe has extensive experience with mentorship and lifestyle/behavioral interventions for minority populations.
- Moderate: Roles of each mentor for project and training are clear.

Weaknesses

- None identified.

5. Environment and Institutional Commitment to the Candidate:

Strengths

- Major: Excellent research environment with significant resources to support this investigator and the proposed aims.
- Major: Institutional commitment is strongly demonstrated at the departmental and institutional levels.

Weaknesses

- None identified.

Protections for Human Subjects:

Acceptable Risks and Adequate Protections

Data and Safety Monitoring Plan (Applicable for Clinical Trials Only):

Acceptable

Inclusion of Women, Minorities and Children:

- Sex/Gender: Distribution justified scientifically.
- Race/Ethnicity: Distribution justified scientifically.
- For NIH-Defined Phase III trials, Plans for valid design and analysis: Not applicable.
- Inclusion/Exclusion of Children under 18: Excluding ages <18; justified scientifically.

Vertebrate Animals:
Not Applicable (No Vertebrate Animals)

Biohazards:
Not Applicable (No Biohazards)

Training in the Responsible Conduct of Research:
Acceptable

Select Agents:
Not Applicable (No Select Agents)

Resource Sharing Plans:
Not Applicable (No Relevant Resources)

Authentication of Key Biological and/or Chemical Resources:
Acceptable

Budget and Period of Support:
Recommend as Requested

CRITIQUE 2

Candidate: 2
Career Development Plan/Career Goals /Plan to Provide Mentoring: 2
Research Plan: 3
Mentor(s), Co-Mentor(s), Consultant(s), Collaborator(s): 2
Environment Commitment to the Candidate: 2

Overall Impact:
The candidate has the potential to transition as an independent investigator. Her goal is to develop and test a short message service (SMS) intervention to improved type 2 diabetes mellitus control among Chinese Americans. There is sufficient evidence that her academic environment is conducive to success. The proposed mentors have the required expertise, and she has had prior mentoring experience. While the research is achievable, but maybe overly ambitious in the context of career development training.

1. Candidate:

Strengths

- The candidate has adequate early research training and is currently completing postdoctoral training at New York University.
- Her acceptance in the NIMHD Health Disparities Institute suggests her credentials make her very competitive among her peers.
- She was also chosen to receive an NYU Outstanding Post-Doc Award.
- She is well-published, with 17 published/accepted manuscripts.

Weaknesses

- None noted.

2. Career Development Plan/Career Goals & Objectives/Plan to Provide Mentoring:

Strengths

- Branching off to establish new within institution collaborations.
- Well-developed training effort.

Weaknesses

- There is some concern that the “experiential immersion” related to CBPR may not be adequate.
- In the context of a very ambitious research plan, there is some concern that the candidate’s career development may suffer.

3. Research Plan:

Strengths

- Theoretically sound.
- Creative (yet, not novel) approach to developing an intervention for an understudied population.

Weaknesses

- Somewhat diffuse; seems overwhelming, with too many components.
- Lack of details related to analysis for Aim 2.
- The CBPR component of the research strategy is weak. While establishment of a community advisory board is a traditional approach, it is primarily for advice on engagement, recruitment, retention, and feedback on intervention development. Lacks true community input.

4. Mentor(s), Co-Mentor(s), Consultant(s), Collaborator(s):

Strengths

- Drs. Trinh-Shevrin and Ogedegbe are well known in the areas of community-based participatory research with Asian Americans.
- All mentors have complimentary expertise and have funded research.

Weaknesses

- None noted.

5. Environment and Institutional Commitment to the Candidate:

Strengths

- The research environment is adequate to support the candidate's career development goals and proposed research.

Weaknesses

- None noted.

Protections for Human Subjects:

Acceptable Risks and Adequate Protections

Data and Safety Monitoring Plan (Applicable for Clinical Trials Only):

Acceptable

Inclusion of Women, Minorities and Children:

- Sex/Gender: Distribution justified scientifically.
- Race/Ethnicity: Distribution justified scientifically.
- For NIH-Defined Phase III trials, Plans for valid design and analysis: Not applicable.
- Inclusion/Exclusion of Children under 18: Excluding ages <18; justified scientifically.

Vertebrate Animals:

Not Applicable (No Vertebrate Animals)

Biohazards:

Not Applicable (No Biohazards)

Training in the Responsible Conduct of Research:

Acceptable

Comments on Format (Required):

- Adequate.

Comments on Subject Matter (Required):

- Adequate.

Comments on Faculty Participation (Required; not applicable for mid- and senior-career awards):

- Adequate.

Comments on Duration (Required):

- Adequate.

Comments on Frequency (Required):

- Adequate.

Select Agents:

Not Applicable (No Select Agents)

Resource Sharing Plans:

Acceptable

Authentication of Key Biological and/or Chemical Resources:

Not Applicable (No Relevant Resources)

Budget and Period of Support:

Recommend as Requested

CRITIQUE 3

Candidate: 2
Career Development Plan/Career Goals /Plan to Provide Mentoring: 2
Research Plan: 3
Mentor(s), Co-Mentor(s), Consultant(s), Collaborator(s): 1
Environment Commitment to the Candidate: 1

Overall Impact:

Dr. Hu's study, "A mobile health intervention to reduce diabetes disparities in Chinese Americans" is an innovative study and strong application with few weaknesses. The candidate has a goal of developing a community-informed SMS intervention to improve diabetes outcomes among Chinese Americans. The candidate has a mentoring team with considerable resources, track record of leading R01 funded studies and career training programs, and expertise in interventions, community-based research, and novel analytic methods. The research aims are sound and achievable but are ambitious in terms of achieving aim 1 in less than 2 years to then prepare for the R00 phase (subsequent aims). Candidate has a strong team and training and program plan to successfully execute the proposed aims and transition to an independent research career. The one core weaknesses noted is the manner in how exactly the community-based approaches will inform each stage of the project.

1. Candidate:

Strengths

- Candidate has an impressive research and publication record, numerous awards and active participation in the larger research community.
- The candidate has support for protected time to conduct training and research activities.

Weaknesses

- None.

2. Career Development Plan/Career Goals & Objectives/Plan to Provide Mentoring:

Strengths

- Candidate has a balance of formal coursework, research immersion, individual and team mentoring meetings and guidance, and direct consultation and technical assistance in research methods and approaches; including community-based research and intervention design.
- In addition to training specific to the research goal, the candidate has a solid plan for professional development and career preparation through a Postdoctoral Professional Development Program.

Weaknesses

- Given the research aims, this training plan seems quite ambitious, but it is balanced with a strong team that the candidate is already working with and fully integrated.
- Didactic coursework and training in intervention design comes at the end of year 2 when the feasibility study should already be underway.

3. Research Plan:

Strengths

- Ambitious research plan that allows for formative research to inform a feasibility trial.
- Preliminary research and similar approach in different population that will be used to development intervention, making this more feasible to accomplish in the proposed timeline.
- New development of intervention for population that has been understudied.

Weaknesses

- In the adaptation of the SMS texts and videos, more detail us needed of the process of how this will be adapted, the theoretical underpinnings or prior work that would enhance the tailoring process.
- What will be your approach or the specific areas of focus during the focus groups with respect to barriers and facilitators? What are the specific ways in which this information will be probed- seems very critical but not much detail.
- Analysis - would be helpful to see models and more detail of explicit models (aim 2).
- Some places say convenience sample, and some say random sample of participants for pilot study.

4. Mentor(s), Co-Mentor(s), Consultant(s), Collaborator(s):

Strengths

- Very strong mentoring team with senior members, center directors, with research and resources to support the candidate in the training aims and research goals.
- Primary and co-mentors with track record of funding, mentoring students and developing junior researchers.

Weaknesses

- None.

5. Environment and Institutional Commitment to the Candidate:

Strengths

- Several division and centers with core support and resources related to disparities among Chinese Americans, behavioral health, interventions and mhealth.

Weaknesses

- None.

Protections for Human Subjects:

Acceptable Risks and Adequate Protections

- Acceptable.

Data and Safety Monitoring Plan (Applicable for Clinical Trials Only):

Acceptable

  - Indicates a Data and Safety Monitoring Board and Plan will be in place for the duration of the study.

Inclusion of Women, Minorities and Children:

- Sex/Gender: Distribution justified scientifically.
- Race/Ethnicity: Distribution justified scientifically.
- For NIH-Defined Phase III trials, Plans for valid design and analysis:
- Inclusion/Exclusion of Children under 18: Excluding ages <18; justified scientifically.

Vertebrate Animals:

Not Applicable (No Vertebrate Animals)

Biohazards:

Not Applicable (No Biohazards)

Training in the Responsible Conduct of Research:

Acceptable

Comments on Format (Required):

- Acceptable.

Comments on Subject Matter (Required):

- Acceptable.

Comments on Faculty Participation (Required; not applicable for mid- and senior-career awards):

- Acceptable.

Comments on Duration (Required):

- Acceptable.

Comments on Frequency (Required):

- Acceptable.

Select Agents:

Not Applicable (No Select Agents)

Resource Sharing Plans:

Not Applicable (No Relevant Resources)

Authentication of Key Biological and/or Chemical Resources:

Not Applicable (No Relevant Resources)

Budget and Period of Support:

Recommend as Requested

THE FOLLOWING SECTIONS WERE PREPARED BY THE SCIENTIFIC REVIEW OFFICER TO SUMMARIZE THE OUTCOME OF DISCUSSIONS OF THE REVIEW COMMITTEE, OR REVIEWERS' WRITTEN CRITIQUES, ON THE FOLLOWING ISSUES:

PROTECTION OF HUMAN SUBJECTS: ACCEPTABLE

INCLUSION OF WOMEN PLAN: ACCEPTABLE

INCLUSION OF MINORITIES PLAN: ACCEPTABLE

INCLUSION OF CHILDREN PLAN: ACCEPTABLE

COMMITTEE BUDGET RECOMMENDATIONS: The budget was recommended as requested.

Footnotes for 1 K99 MD012811-01; PI Name: Hu, Lu

NIH has modified its policy regarding the receipt of resubmissions (amended applications). See Guide Notice NOT-OD-14-074 at http://grants.nih.gov/grants/guide/notice-files/NOT-OD-14-074.html. The impact/priority score is calculated after discussion of an application by averaging the overall scores (1-9) given by all voting reviewers on the committee and multiplying by 10. The criterion scores are submitted prior to the meeting by the individual reviewers assigned to an application, and are not discussed specifically at the review meeting or calculated into the overall impact score. Some applications also receive a percentile ranking. For details on the review process, see http://grants.nih.gov/grants/peer_review_process.htm#scoring.

MEETING ROSTER

The roster for this review meeting is displayed as an aggregated roster that includes reviewers from multiple MD Special Emphasis Panels of the Aggregate Roster for NIMHD Special Emphasis Panels for the 2018/05 council round.
This roster for MD is available at:
http://public.era.nih.gov/pubroster/Reports?DOCTYPE=SEP&DESFORMAT=PDF&AGENDA_SEQ_NUM_P=338507
